# Dexmedetomidine Mediates Neuroglobin Up-Regulation and Alleviates the Hypoxia/Reoxygenation Injury by Inhibiting Neuronal Apoptosis in Developing Rats

**DOI:** 10.3389/fphar.2020.555532

**Published:** 2020-10-07

**Authors:** Yan Gao, Yongfang Zhang, Yunxia Dong, Xiuying Wu, Hongtao Liu

**Affiliations:** ^1^Department of Anesthesiology, The First Affiliated Hospital of Hebei North University, Zhangjiakou, China; ^2^Department of Anesthesiology, Shengjing Hospital of China Medical University, Shenyang, China

**Keywords:** dexmedetomidine, neuroglobin, hypoxia/reoxygenation injury, neonatal hypoxic brain injury, neuropharmacology

## Abstract

**Background:**

Exploring the effective therapy for neonatal hypoxic-ischemic brain injury is an important goal. This study was designed to investigate how dexmedetomidine (DEX) contribute to hypoxic brain injury.

**Methods:**

Developing Sprague-Dawley rat models of hypoxia/reoxygenation (H/R) injury were constructed to simulate neonatal hypoxic brain injury for DEX treatment. Immunohistochemistry and western blot were performed to measure neuroglobin (Ngb) protein expression in hippocampal tissues. Hippocampal neuron injury and apoptosis were detected by Nissl staining and TUNEL assay, respectively. A Morris water maze (MWM) test was performed to evaluate the long-term learning and memory function.

**Results:**

The expression of Ngb was increased following H/R model establishment and up-regulated by medium and high doses of DEX, but not up-regulated by low doses of DEX. Medium and high doses of DEX alleviated the H/R injury as well as induced the reduction of Nissl bodies and apoptosis. Besides, medium and high doses of DEX down-regulated cytosolic Cyt-c, Apaf-1, and caspase-3 in H/R injury model. MWM test showed that medium and high doses of DEX significantly shortened the escape latency and enhanced the number of platform crossings. However, low doses of DEX have no effect on Nissl bodies, mitochondrial apoptosis, expression of apoptosis-related proteins and long-term learning functions.

**Conclusions:**

DEX induced Ngb expression in H/R rat models. The neuroprotection of DEX-mediated Ngb up-regulation may be achieved by inhibiting neuronal apoptosis through the mitochondrial pathway. Findings indicated that DEX may be useful as an effective therapy for neonatal hypoxic brain injury.

## Introduction

Neonatal hypoxic-ischemic (H/I) brain injury is a frequently encountered clinical problem in survivors of perinatal asphyxia, which is associated with mortality and neurodevelopmental morbidity (Cheng et al., 1997). Due to the selective regional and cellular vulnerability, it usually presents different outcomes in neonates (Cowan et al., 2003). Neonatal H/I cerebral injury often caused multiple adverse neurologic outcomes, including cognitive disorder, epilepsy, cerebral palsy, and mental retardation (van Handel et al., 2007). Statistically, the incidence of asphyxia is approximately 0.2% in full-term or near full-term neonates, and has reached to 60% in low-birth-weight or premature neonates (Alibrahim et al., 2013). However, there is currently no effective therapy for neonatal H/I cerebral injury (Perlman, 2006). Thus, exploring the therapeutic targets and effective therapies for the H/I brain injury is an important goal in clinical.

Dexmedetomidine (DEX) is a selective α_2_ adrenergic agonist that has been used clinically for sedation, analgesia, and anxiolysis with much less respiratory depression than other sedatives (Walker et al., 2006). Dex also wears off quickly and does not live people drowsy. Previously, DEX has been reported to produce its neuroprotective effect *via* the α_2A_-adrenoceptor subtype (Ma et al., 2004). DEX could attenuate the massive release of catecholamines which occur with cerebral hypoxic-ischemia in multiple parts of the brain (Matsumoto et al., 1993). The use of DEX has been described widely in neonates and infants (Potts et al., 2009; Ezzati et al., 2014). However, the risks of adverse effects (bradycardia, hypotension and hypertension) from DEX were also been reported, particularly with increasing plasma concentrations (Mason and Lerman, 2011; Ezzati et al., 2017). Therefore, close monitoring of circulatory dynamics and careful dose titration of DEX have been recommended (Ezzati et al., 2014). Although the neuroprotective effects of DEX have been demonstrated extensively (Sifringer et al., 2015), the molecular mechanism and mediated signaling pathways of DEX remain to be elucidated. Additional mechanisms for the neuroprotective effects of DEX are likely to exist. Understanding the molecular mechanism of DEX induced-neuroprotective effects would be beneficial for the future development of pharmacotherapy in extending the therapeutic window.

Neuroglobin (Ngb) is a newly discovered vertebrate, monomeric globin with abundant expression in neurons (Burmester et al., 2000). It has high oxygen affinity and preferential localization to vertebrate brain, thus increasing the availability of oxygen to brain tissue (Zhu et al., 2017). Recently, it is demonstrated that Ngb is overexpressed in H/I injury model and has protective effect on nerve cells from apoptosis, thereby protecting the neurons from H/I injury (Sun et al., 2001; Dong et al., 2010). Ngb plays an important role in neuroprotection by improving mitochondria function and decreasing oxidative stress (Liu et al., 2009). Interestingly, Jin et al. previously reported that many drugs, such as deferoxamine, short-chain fatty acids cinnamic acid and valproic acid (VPA), could induce the expression of Ngb (Jin et al., 2011). Thus, we speculated that the neuroprotective effects of DEX may have a potential association with Ngb, in which the DEX may induce the expression of Ngb to play an active role in its neuroprotection for hypoxic brain injuries. Thus, in this study, developing rat models of hypoxia/reoxygenation (H/R) injury were constructed to simulate the neonatal hypoxic brain injury. Rats were exposed to different doses of DEX to evaluate the potential mechanisms by which DEX can contribute to neuroprotection of the neonatal brain following H/R.

### Materials and Methods

#### Animals

Total of 36 Sprague-Dawley (SD) rats (12 males and 24 females, weight 200–260 g) were purchased from Liaoning Changsheng Biotechnology co. LTD (Liaoning, China). All rats were housed at room temperature (24 ± 1°C), a 14:10 light/dark cycle (ie, 14 h of light per 24 h), and free access to food and water. All animal protocols were carried out in accordance with Regulations for the Administration of Affairs Concerning Experimental Animals of People’s Republic of China and approved by the Ethics Committee of our hospital.

Two females were introduced to each individually caged male. After the female rats are confirmed pregnant, the rats were removed to fresh cages until natural delivery. The date of birth for the neonatal rat pups was defined as 0-day-old (P0). In order to reduce the difference caused by nutrient imbalance on feeding, if there are more than 10 rats per nest, the extra rats were randomly removed to keep 10 rats per nest. Total of 170 7-day-old (P7) male and female rats (weight, 12–15 g) were randomly selected from the neonatal rat pups.

#### H/R Model and DEX Treatment

All P7 rats were randomly divided into five groups (n=34 per group): normal vehicle control group (C group), hypoxia/reoxygenation model group (H/R group), low dose of DEX treatment at 25 μg/kg body weight (D1 group), middle dose of DEX treatment at 50 μg/kg body weight (D2 group), and high dose of DEX treatment at 75 μg/kg body weight (D3 group). 136 rats in H/R, D1, D2 and D3 groups were used to establish the H/R model as described elsewhere (Gao et al., 2019). Briefly, rats were placed into the closed hypoxic container and exposed to hypoxia with 8% O_2_/92% N_2_ gas mixture for 120 min. Then N_2_ supply was interrupted, followed by reoxygenation with 50% O_2_ for 30 min. Subsequently, rats in D1, D2 and D3 groups were intraperitoneally injected with 25 μg/kg, 50 μg/kg, and 75 μg/kg DEX (dissolved in normal saline, Hengrui Medicine, Jiangsu, China) respectively. Meanwhile, rats in control group were intraperitoneally injected with equal amount of normal saline. The used concentration gradient of DEX was determined according to previous studies (Li et al., 2014; Pancaro et al., 2016). P7 rats were placed in an incubator maintained at 37°C until waking up, then transferred to their mother for natural feeding.

#### Preparation of Hippocampal Slices

Three rats from each group were anesthetized with intraperitoneal injection of chloral hydrate at 2 h, 24 h, 48 h and 72 h after treatment. Then, rats were perfused by intracardiac perfusion with 10 ml normal saline followed by 20 ml 4% paraformaldehyde. The brains were removed and fixed in 4% paraformaldehyde, embedded into paraffin and then prepared into hippocampal slices (4 μm). Hippocampal slices were used for the following analyses, including immunohistochemistry (IHC), Nissl staining and terminal deoxynucleotidyl transferase-mediated dUTP nick end-labeling (TUNEL) assay.

#### IHC Analysis

A standard streptavidin-peroxidase-biotin method was performed for IHC staining (Zhang et al., 2013). Paraffin sections (4 μm) were deparaffinized in xylene, rehydrated in graded ethanol, and washed in phosphate buffer (PBS). Then sections were treated with sodium citrate buffer (10 mM, pH 6.0) for 7 min for unmasking epitopes. Subsequently, endogenous peroxidase activity was blocked with 3% H_2_O_2_ for 30 minutes, followed by incubation goat serum to block nonspecific staining. Sections were incubated with primary antibody against Ngb (N7162, dilution 1:200, Sigma-Aldrich, St Louis. MO USA) overnight at 4°C, followed by incubation with the biotinylated goat anti-rabbit secondary antibody for 20 min. Then, sections were incubated with horse radish peroxidase (HRP)-conjugated streptavidin-biotin complex for 30 min. Detection was performed using diaminobezidin (DAB, Dako, Denmark) as chromogen. Images were collected at 400× magnification under Olympus BX51 Epi-fluorescent microscopy (Olympus Co. Tokyo, Japan). Positive staining was defined as >10% of cells appearing as brown granules.

#### Western Blot

Another three rats from each group were anesthetized with intraperitoneal injection of chloral hydrate at 2 h, 24 h, 48 h and 72 h after treatment. Hippocampal tissues were isolated form the brain on ice and stored in a refrigerator at −80°C until used. Western blot assay was performed according to the standard protocol (Sen et al., 2019). For western blotting of cytosolic cytochrome c (Cyt-c), cytosolic fractions were firstly isolated using cytosol/mitochondria fractionation kit (Beyotime, China) according to the manufacturer’s protocol. Proteins were isolated by using ice-cold RIPA lysis buffer (Beyotime Biotechnology, China) and quantified using bicinchoninic acid (BCA) method with BSA as standard according to the manufacturer’s protocol (Beyotime Biotechnology, China). Then, equal amounts of proteins (40 µg) of each sample were separated on 10% sodium dodecyl sulfate-polyacrylamide gel electrophoresis (SDS-PAGE) and was transferred onto polyvinylidene fluoride (PVDF) membrane (Millipore, USA). Then PVDF membrane was blocked in 5% (W/V) skim milk at room temperature for 2 h. Subsequently, PVDF membrane was incubated at 4°C with specific primary antibodies against to Ngb (1:1000 dilution, GTX54552, GeneTex), Cyt-c (1:500 dilution, 10993-1-AP, Proteintech, China), Apaf-1 (500 dilution, 201710-1-AP, Proteintech, China), caspase-3 (1:1000 dilution, 9662S, CST) overnight. GAPDH was used as internal control. After washing thrice with Tris buffer saline with 0.1% Tween 20, PVDF membrane was incubated with corresponding HRP-linked goat anti-rabbit IgG antibody (1:5000 dilution, Zhongshan Golden Bridge Biotechnology Co., Ltd., China) for 1.5 h at room temperature. Bands were visualized using the enhanced chemiluminescence kit (Thermo, USA) and the band intensity of western blot was semi-quantified subsequent to normalization with the density of internal control using Image J software (National Institutes of Health, USA).

#### Nissl Staining

Nissl staining was performed using a Nissl Staining Solution kit (Solarbio, USA). Paraffin sections were deparaffinized in xylene, stained with cresyl violet stain for 1 h at 56°C and washed with deionized water. Then, sections were placed in Nissl Differentiation Solution for 2 min. All stained sections were progressively dehydrated in a graded series of 70%, 80%, 90%, and finally 100% ethanol, cleared in xylol for 15 min and finally fixed with neutral balsam. The sections were visualized by a light microscope (Olympus, Tokyo, Japan).

#### TUNEL Assay

TUNEL assay using One Step TUNEL Apoptosis Assay Kit (Roche Applied Science, USA) was performed to evaluate the cell apoptosis according to manufacturer’s protocol. In brief, paraffin sections were deparaffinized in xylene, and incubated in PBS for 5 min and proteinase K (20 μg/ml) for 12 min at 37°C, respectively. Then sample was rinsed three times with PBS for 5 min each time and incubated in 0.1% Tritol at room temperature for 10 to 30 min. After washing with PBS, sample was incubated with TUNEL agents in a cassette at 4°C overnight. Finally, cells were counterstained with DAPI, and visualized under a fluorescence microscope (Olympus, Tokyo, Japan). Percentage of cell apoptosis as calculated according to the following formula: Apoptosis rate=positive cell count/all cell count.

#### Morris Water Maze (MWM) Tests

The MWM tests were performed to evaluate the long-term learning and memory functions. Ten rats in each group received the MWM tests for 6 consecutive days from 28 days after treatment. Rats were trained in a pool (diameter = 160 cm, height = 60 cm) with a 10 cm diameter platform 2 cm above the water surface. Water temperature was maintained at 23 ± 1°C. The MWM paradigm consists of five consecutive learning days with four 90-second trials per day from different quadrants. At the end of the trial, either when the rat had found the platform or when 90 s had elapsed, rats were allowed to rest on the platform for 15 s. On day 6, the platform was removed and mice were allowed to swim freely for 90 s. Ethovision^®^ XT video tracking system (Noldus, Wageningen, Netherlands) recorded and analyzed the movement data, including escape latency, swim path, distance and average speed.

#### Statistical Analysis

Statistical analysis was performed with IBM SPSS statistics 20.0 (SPSS Inc., USA). Results are expressed as mean ± the standard error of the mean (SEM) of three separate experiments. We tested the normality of the data distribution with Kolmogorov-Smirnov, the distribution of the data was considered normal. Statistical significance between multiple groups was determined with one-way analysis of variance (ANOVA) followed by a Tukey’s *post hoc* test. Statistical significance of the escape latency between multiple groups was determined with two-way ANOVA. Differences were considered statistically significant when *p*<0.05.

### Results

#### DEX Mediates the Up-Regulation of Ngb in H/R Injury

**Figure 1:**
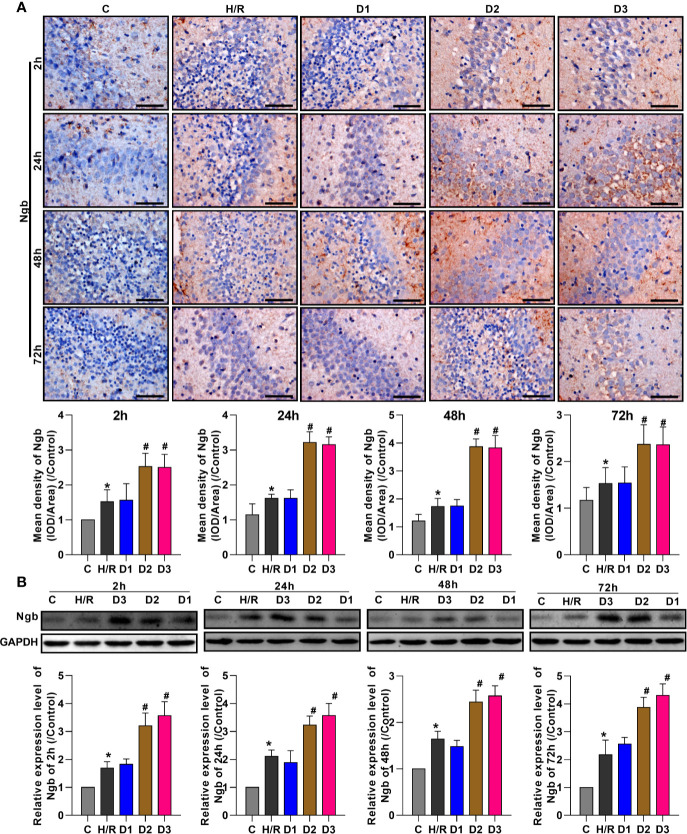
DEX mediates the up-regulation of neuroglobin in hypoxia/reoxygenation injury. **(A)** Immunohistochemical analysis and **(B)** western blot assay were respectively performed at 2, 24, 48, and 72 h after H/R model establishment to detect the neuroglobin expression in hippocampal tissues. Scale bar: 100 μm; Magnification: 400×. Data are expressed as mean ± SEM of three rats per group at each time point. *p < 0.05, compared to control; ^#^*p* < 0.05, compared to H/R group.

To evaluate the effects of DEX on Ngb expression, the expression levels of Ngb in hippocampal tissue were analyzed at different times using IHC and western blot, respectively. IHC analysis showed that the Ngb expression was up-regulated in H/R injury rat model at each time point compared to the control (**Figure 1A**). After different doses of DEX exposure, medium (50 μg/kg) and high doses (75 μg/kg) of DEX significantly up-regulated the Ngb expression compared to the H/R group, while low doses (25 μg/kg) of DEX had no effect on the expression of Ngb (**Figure 1A**). Consistently, similar results were observed in the western blot analysis (**Figure 1B**). Overall, these results indicated that Ngb may participate in the H/R injury, and DEX mediated the up-regulation of Ngb in H/R injury when achieved a certain effective dose.

#### DEX-Mediated Ngb Up-Regulation Alleviates the Hippocampal Neuron Injury

**Figure 2:**
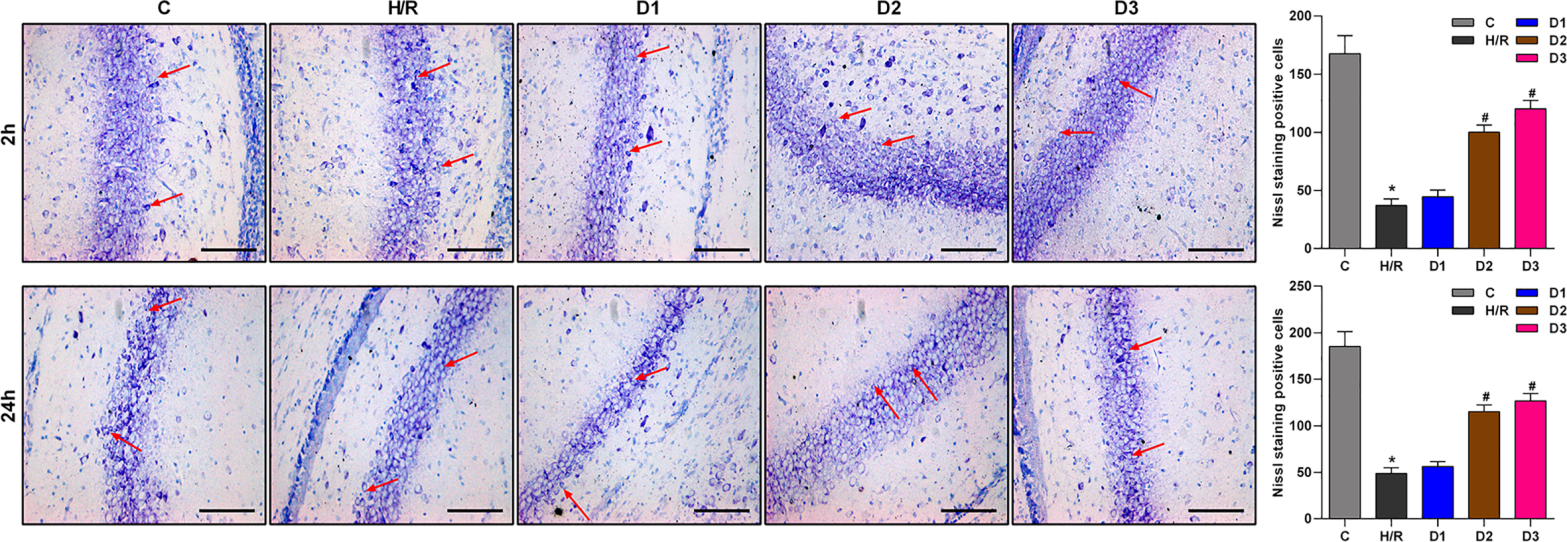
Nissl staining results of hippocampal CA1 region. The red arrows show Nissl bodies in the hippocampal CA1 regions. Nissl staining was performed at 2 and 24 h after H/R model establishment to evaluate the hippocampal neuron injury. Data are expressed as mean ± SEM of three rats per group at each time point. Scale bar: 50 μm. Magnification: 200×.

As previously reported, Ngb has neuroprotective effects. Thus, we speculated that DEX-mediated Ngb up-regulation may affect the hippocampal neuron injury. To assess the neuroprotective effects of DEX-mediated Ngb up-regulation, Nissl staining was performed. As shown in **Figure 2**, Nissl bodies in the hippocampal CA1 region appeared as blue granules or plaques (red arrow). In the control group, neurons had abundant Nissl bodies. In the H/R model group, Nissl bodies reduced, disintegrated and even disappeared, and the number of neurons decreased compared to the control group, which suggested that hippocampal neurons were injured after H/R model establishment. When H/R model treated with low doses of DEX, Nissl bodies still reduced and disintegrated with small amount of neurons. When H/R model treated with medium and high doses of DEX, Nissl bodies reappeared and increased, meanwhile the number of neurons increased compared to the H/R group, and neurons showed a granular distribution of Nissl bodies. These results suggested that the H/R injury was recovered by medium and high doses of DEX. Overall, these results indicated that DEX-mediated Ngb up-regulation alleviates the hippocampal neuron injury in H/R injury.

#### DEX-Mediated Ngb Up-Regulation Inhibits Mitochondrial Apoptosis of Hippocampal Neurons in Developing Rat Model of H/R Injury

**Figure 3:**
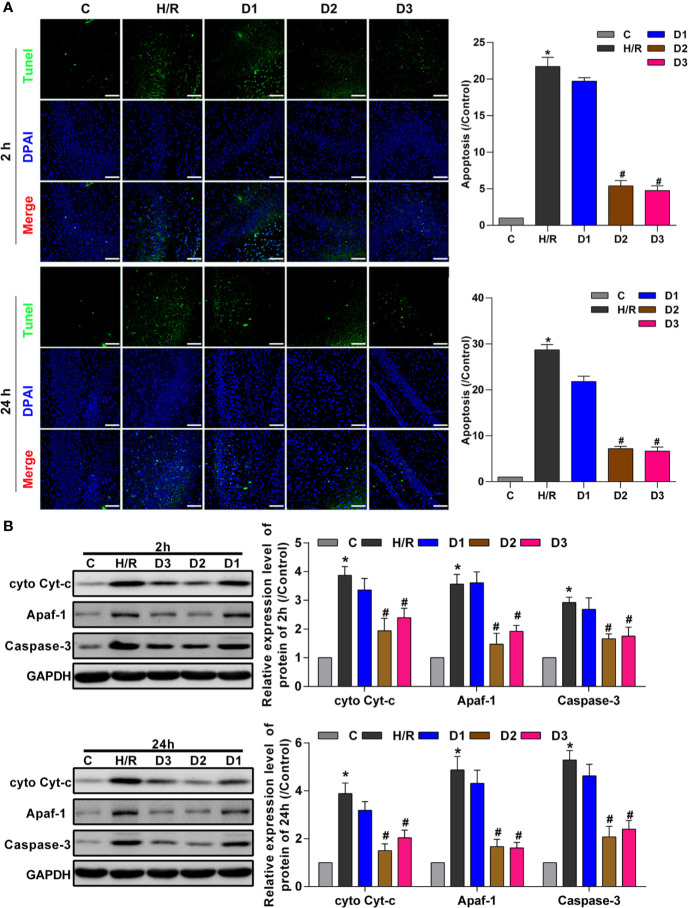
DEX-mediated Ngb up-regulation inhibits mitochondrial apoptosis of hippocampal neurons. **(A)** TUNEL assay results of hippocampal tissues at 2 and 24 h after H/R model establishment to evaluate apoptosis. **(B)** Western blot assay was used to detect the expression of cytosolic Cyt-c, Apaf-1, and caspase-3 at 2 and 24 h after H/R model establishment. Scale bar: 100 μm. Magnification: 200×. Data are expressed as mean ± SEM of three rats per group at each time point. *p < 0.05, compared to control; ^#^*p* < 0.05, compared to H/R group.

To confirm the neuroprotective effects of DEX-mediated Ngb up-regulation, TUNEL assay was performed to detect the hippocampal neuron apoptosis. After H/R model establishment, cell apoptosis rate was enhanced compared with the control (**Figure 3A**). Although low doses of DEX had no effect on cell apoptosis, medium and high doses of DEX significantly inhibited the cell apoptosis at each time point (**Figure 3A**). Mitochondrial apoptosis is characterized by the release of Cyt-c from mitochondria into the cytoplasm (Ezzati et al., 2014), thus we detect the expression of apoptosis-related proteins. As shown in **Figure 3B**, the expressions of cytosolic Cyt-c, apoptotic protease activating factor-1 (Apaf-1), and caspase-3 increased significantly in H/R group compared to the control. After low dose of DEX exposure, the expressions of cytosolic Cyt-c, Apaf-1, and caspase-3 were not changed obviously. When H/R model treated with medium and high doses of DEX, the expressions of cytosolic Cyt-c, Apaf-1, and caspase-3 were significantly inhibited compared to the H/R group (**Figure 3B**). Overall, these results indicated that DEX-mediated Ngb up-regulation inhibits mitochondrial apoptosis of hippocampal neurons in H/R injury.

#### DEX-Mediated Ngb Up-Regulation Improves the Long-Term Learning and Memory Function in Developing Rat Model of H/R Injury

**Figure 4:**
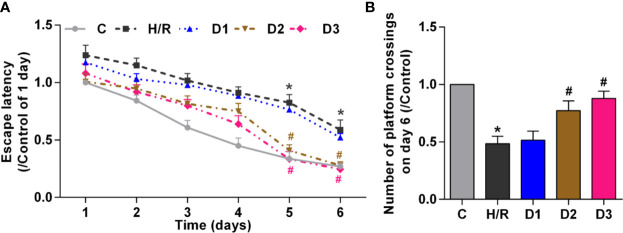
DEX-mediated Ngb up-regulation improves the long-term learning and memory functions. Ten rats in each group received the MWM tests for six consecutive days from 28 days after H/R model establishment. **(A)** The escape latency from day 1 to day 6. **(B)** The number of platform crossings on day 6. Data are expressed as mean ± SEM of 10 rats per group. *p < 0.05, compared to control; ^#^*p* < 0.05, compared to H/R group.

To verify the effects of DEX-mediated Ngb up-regulation on long-term learning and memory functions in developing rat model of H/R injury, MWM tests was performed on the 28th day of neonatal rats. On the five learning days, the mean escape latency for the trained rat model of H/R injury to find the hidden platform increased compared to the control (**Figure 4A**), and the number of platform crossings on day 6 decreased obviously (**Figure 4B**). Low doses of DEX exposure had no effect on the escape latency and the number of platform crossings, while medium and high doses of DEX treatment significantly shorten the escape latency and enhanced the number of platform crossings (**Figure 4**). Overall, these results suggested that H/R injury affect the long-term learning and memory functions in developing rats, and DEX could reverse the dysfunction of learning and memory caused by H/R injury.

### Discussion

Ngb is a recently discovered hypoxia-inducible protein with cytoprotective effects in animal models of Alzheimer’s disease, stroke, myocardial infarction, and other related disorders (Khan et al., 2006). It thus appears that increasing the serum Ngb levels might have therapeutic benefit for some disorders. However, at present, the direct administration of Ngb is impractical in clinical due to the inability of mammalian Ngb to penetrate cell membranes (Haines et al., 2012). Thus, exploring some drugs that can enhance Ngb expression has a vital significance. Notably, a few small molecule drugs have been identified which can enhance Ngb expression (Zhu et al., 2002; Jin et al., 2011). Nevertheless, there are few studies on the relationship of DEX and Ngb. Accordingly, we focused on the effects of DEX on Ngb in the developing rat model of H/R injury for the first time. In the present study, we found that Ngb was up-regulated in H/R injury model, and then DEX could further induce the up-regulation of Ngb expression. Moreover, the results demonstrated that DEX could alleviate the hippocampal neuron injury, inhibit mitochondrial apoptosis of hippocampal neurons and improve the long-term learning and memory functions in developing rat model of H/R injury.

DEX, as a selective α_2_ adrenergic agonist, has high specificity and selectivity. Due to its shorter half-life period and good controllability, DEX became a convenient and desirable drug (Zientara et al., 2019). Besides, DEX has known to attenuate isoflurane-induced neurocognitive impairment, acetaminophen-induced liver injury, myocardial and hepatic ischemia-reperfusion (I/R) injury *via* various signaling pathway (Sanders et al., 2009; Chou et al., 2019; Geng et al., 2019). However, the molecular mechanism of the neuroprotective effect of DEX has not been adequately illuminated. Therefore, to clarify the mechanism of the neuroprotective effect of DEX, we firstly detected the Ngb expression after DEX exposure in H/R injury model. Fortunately, we observed that the medium and high doses of DEX significantly up-regulated the Ngb expression in H/R injury model at each time point. The result suggested that Ngb expression could be induced pharmacologically by DEX when achieved a certain effective dose. Similarly, previous reports have also reported that some small molecules like deferoxamine (Sun et al., 2001), hemin (Zhu et al., 2002; Song et al., 2014), cinnamic acid and VPA (Jin et al., 2011) have capable of inducing Ngb expression in neurons. Nevertheless, the previous studies have not illuminated whether the induction of Ngb contributes to their protective actions (Jin et al., 2011). In our preliminary study, we also found that DEX at a certain dose could up-regulate the expression of Ngb and protect hippocampal neurons against H/R-induced apoptosis through activating HIF-1α/p53 signaling (Gao et al., 2019). In addition, we found that DEX could induce the difference of Ngb expression after 2 hours. One possible explanation is that DEX is a strong and fast-acting α_2_ adrenergic receptor agonist, which is capable of causing the difference protein expression in a short time. Taken together, we demonstrated that Ngb can be induced by DEX, and that this occurs *in vivo* when achieved a certain effective dose. We speculated that the neuroprotective effects of DEX might be act through the DEX-mediated Ngb up-regulation.

To verify whether DEX-mediated Ngb up-regulation has neuroprotective effects, we further detected the hippocampal neuron injury, neuronal apoptosis and the long term learning and memory functions in H/R injury model. As expected, DEX-mediated up-regulation of Ngb alleviates the H/R injury in developing rats. The hypoxic-ischemia could cause cytochrome c (Cyt-c) release from mitochondria into the cytoplasm and thereby to initiate the endogenous apoptosis pathway (Borutaite et al., 2001; Potts et al., 2009). Once Cyt-c was released into the cytoplasm, it would bind to Apaf-1 in the presence of dATP, resulting in the apoptosome formation. And then, the apoptotic body facilitates activation of caspase-9 and caspase-3, which in turn triggers the cascade reactions leading to apoptosis (Borutaite et al., 2003). Conversely, the inhibition of the formation of apoptosome would contribute neurons to withstand mitochondrial damage (Gama et al., 2014). There exists a very facile redox reaction between Cyt-c and Ngb, and the Cyt-c could bind Ngb to form Cyt-c/Ngb complex thereby to suppress apoptosis (Brittain et al., 2010; Raychaudhuri et al., 2010). When Cyt-c/Ngb complex was decomposed, apoptotic body increased to promote the apoptosis and neuron damage. In a word, the certain serum levels of Ngb could interact with Cyt-c to inhibit endogenous apoptosis, thus playing its neuroprotective effect (De Marinis et al., 2011). In present study, we found that the medium and high doses of DEX promoted the expression of Ngb, and further inhibited the expression of cytosolic Cyt-c, Apaf-1, and caspase-3 and the mitochondrial apoptosis. However, the low dose of DEX has no effect both on the Ngb expression and expression of cytosolic Cyt-c, Apaf-1, and caspase-3. Thus, we speculated that DEX could induce Ngb expression and in turn inhibit neuronal apoptosis. The neuroprotective effects of DEX-mediated Ngb up-regulation may be related to the maintenance of mitochondrial function.

Additionally, MWM test showed that the escape latency and number of platform crossings were improved by medium and high doses of DEX treatment significantly, but not low dose of DEX. Our findings were similar with these previous researches (Gao et al., 2016; Shan et al., 2018). Previously, Xin et al. have reported that DEX ameliorates electroconvulsive therapy-induced learning and memory impairments in depressed rats *via* the NR2B-ERK signaling cascade (Gao et al., 2016). Together with other evidence, it can be speculated that DEX-mediated Ngb up-regulation could reverse the dysfunction of learning and memory caused by H/R injury.

It was noting that bradycardia, hypotension, and hypertension have been observed in children to varying degrees, depending on the plasma concentration of DEX (Potts et al., 2008; Mason and Lerman, 2011). Meanwhile, it is previously reported that DEX induce cerebral hypoperfusion and neurotoxicity in small-animal models (Nakano and Okamoto, 2009; Ezzati et al., 2017). In our study, despite obvious drug toxicity and adverse reactions were not observed after DEX treatment, close monitoring of circulatory dynamics and careful dose titration of DEX still needed.

The limitation of present study is that the detailed signaling pathway was not well researched. We hypothesized the mechanism of neuroprotective effects of DEX based only on changes in protein levels, which may not adequately explain the internal mechanism. Thus, the downstream signaling pathway of DEX-mediated Ngb up-regulation should be focused in the further studies. Additionally, the lack of inhibition expression of Ngb was a potential criticism for this study. We would research the neuroprotective mechanism of Ngb by changing the Ngb expression.

In summary, this study investigated the effects of DEX on Ngb expression and the H/R injury in developing rats. It is demonstrated that DEX could induce the Ngb expression pharmaceutically when achieved a certain effective dose. Furthermore, the DEX mediated up-regulation of Ngb and inhibited mitochondrial apoptosis by suppressing cytosolic Cyt-c, Apaf-1, and caspase-3. These findings indicated that DEX may be useful as an effective therapy for the H/I brain injury. The investigations on detailed mechanism of DEX and Ngb in H/R injury remain to be further researched.

## Data Availability Statement

All data generated or analysed during this study are included in this published article.

## Ethics Statement

The animal study was reviewed and approved by the Ethics Committee of Shengjing Hospital of China Medical University.

## Author Contributions

Conceptualization: YZ and YG. Methodology: YG and YD. Formal Analysis: YG. Resources: YD and XW. Data Curation: YG and XW. Writing—Original Draft Preparation: YG. Writing—Review and Editing: HL. Visualization: YG. Supervision: HL. Project Administration: YG. All authors contributed to the article and approved the submitted version.

## Funding

This research was supported by National Natural Science Foundation of China (No. 81271370).

## Conflict of Interest

The authors declare that the research was conducted in the absence of any commercial or financial relationships that could be construed as a potential conflict of interest.
